# Women’s Special Issue Series: Biomedicines

**DOI:** 10.3390/biomedicines12030471

**Published:** 2024-02-20

**Authors:** Letizia Polito

**Affiliations:** Department of Medical and Surgical Sciences-DIMEC, Alma Mater Studiorum—University of Bologna, 40126 Bologna, Italy; letizia.polito@unibo.it

Following the invitation of *Biomedicines*, we decided to accept the project of this Special Issue because we believe that in many situations gender prejudices still exist and put women in a disadvantaged position for the dissemination of their research, preventing the scientific community from benefiting from a plurality of voices in the interpretation of scientific research. The aim of our Special Issue, entitled “Women’s Special Issue Series: Biomedicines” was to celebrate and highlight the achievements of women in the development of new molecules and therapies, particularly regarding new therapeutic targets, therapeutic strategies, and research on biomedicines and biopharmaceutical products. This Special Issue includes nine manuscripts, among which there are seven high-quality research articles, one meta-analysis and systematic review, and one comprehensive review article, published from 2022 to 2023.

The epithelial cell adhesion molecule (EpCAM) has an important role in cancer cellular proliferation, differentiation, adhesion, and migration [1]. The study by Zia et al. [2] proposed that costunolide, a medicinal herb with anticancer properties, could have potential applications for acute lymphoblastic leukemia therapy. The expression of EpCAM and its downstream target genes (Myc and TERT) was downregulated upon treatment with costunolide in Jurkat cells. Costunolide treatment caused a reduced expression of NFκB, a transcription factor of EpCAM, Myc, and TERT, thus suggesting that the drug is capable of inhibiting gene expression by NFκB and its targets.

In patients with diabetes, lower extremity arterial disease (LEAD) is particularly frequent and has a worse prognosis than in patients without diabetes, representing an important risk factor for amputation [3]. In a rat model with LEAD induced by type 2 diabetes mellitus, Shati et al. [4] demonstrated that the induction of femoral artery ultrastructural alterations and the expression of vascular AGE products appeared to be inhibited by the antidiabetic drug metformin throughout the duration of the study (12 weeks). Furthermore, levels of endothelin-1 and nitric oxide synthase were also reduced, as were reduced dyslipidemia and inflammation.

Cyclophosphamide is one of the most common anticancer drugs used in the management of many cancers and autoimmune diseases; in addition to the specific effect on tumor cells, it also produces numerous toxic effects, among which one of the most important is cardiotoxicity [5]. Attia et al. [6] demonstrated the protective role of vitamin E (vit E) in improving cardiotoxicity, induced by cyclophosphamide in rats, lowering serum levels of cardiac damage markers. Moreover, vit E improved the histopathological alterations caused by cyclophosphamide, and no evidence of cardiotoxicity was observed.

Doxorubicin is another important drug used for anti-tumor therapy which, in addition to its effectiveness against tumor cells, also has important side effects, such as toxicity for various tissues and organs [7]. Thus far, many efforts have attempted to reduce the organ toxicity of doxorubicin. The study by Karim et al. [8] examined the various mechanisms through which urolithin A (URO A), a metabolite produced by human colon microbiota [9], protected against doxorubicin-induced liver injury [10] in Wistar rats. The authors demonstrated that URO A reduced doxorubicin-induced liver injury by reducing oxidative stress, inflammation, and apoptosis.

Saporin [11] is a single-chain ribosome-inactivating protein (RIP) [12] that has low toxicity in cells and animals. When the protein is linked to a carrier that facilitates cellular uptake, the protein can become highly and selectively toxic to the cellular target of the carrier. For this reason, saporin is often used to construct immunotoxins or other hybrid conjugates [13]. The article by Bortolotti et al. [14] examined the effect of the most frequently used heterobifunctional reagents on the saporin molecule, with the aim of inserting a chemical bridge between the toxin and the carrier. The authors evaluated the capability of derivatized saporin to maintain its enzymatic properties, i.e., protein synthesis inhibition, deadenylation of DNA, and its biologic properties, i.e., in vitro cytotoxicity. Therefore, this research can be of interest for the construction of saporin-based immunoconjugates, also when small molecules are considered as carriers.

Nanoparticle drug delivery systems are designed to improve the stability/solubility of the cargo, to help its entry into the cell, and to increase its safety and efficacy [15]. Marshall et al. [16] constructed a sodium iodide symporter (NIS)-mediated I-131 radiolabeled polylactic-co-glycolic acid (PLGA) nanoparticle, to evaluate the efficacy of a single dose versus fractionated I-131 doses in a 3D model. The effectiveness of NIS-targeted delivery was demonstrated on MDA-MB-231 cells derived from breast cancer. The nanoparticles successfully delivered fractionated radiotherapeutics. Moreover, the nanoparticles exhibited biocompatibility with blood and had no negative impact on erythrocytes.

Immunotherapy with programmed death 1 (PD-1) and programmed death-ligand 1 (PD-L1)-targeted monoclonal antibodies has strongly modified the therapeutic and prognostic scenario for several malignancies. The FDA approved the assessment of PD-L1 expression as a diagnostic test to evaluate patient eligibility for anti-PD-L1 immunotherapy for the treatment of non-small cell lung carcinoma [17]. The study by Kim et al. [18] investigated PD-L1 expression in endometrial cancer, its connection to prognostic factors, and survival outcomes in 232 patients (31.5% of them had PD-L1-positive tumors). There was no statistically significant difference in progression-free survival and overall survival between patients with PD-L1-positive and PD-L1-negative endometrial cancer. Interestingly, PD-L1 expression was significantly correlated with poor prognostic factors of endometrial cancer, such as histological grade, myometrial invasion, and metastasis.

Numerous studies have identified long non-coding RNAs (lncRNAs) as possible cancer therapy targets or diagnostic and prognostic biomarkers for different types of tumors [19]. The review by Al-Shehri and Bakhashab [20] summarizes the knowledge available so far on oncogenic lncRNAs and their involvement in different tumors, mainly prostate cancer, osteosarcoma, and bone metastasis. The dysregulation of lncRNA expression is implicated in tumor proliferation and metastasis. However, their specific mechanisms and functions in cancer remain not completely understood. Considering oncogenic lncRNAs and their role in prostate cancer, osteosarcoma, and metastasis, and the underlying mechanism, may help to better manage and treat these malignancies.

Fluid therapy is the standard therapy in acute pancreatitis, but the type of fluid to utilize is debated among clinicians [21]. The review and meta-analysis by Ocskay et al. [22] aimed to summarize all the available evidence from randomized controlled trials comparing lactated Ringer’s solution with normal saline used in fluid therapy for acute pancreatitis patients, both adult and pediatric. The authors placed particular emphasis on the clinically relevant results obtained after fluid therapy. Moderate-to-severe acute pancreatitis, mortality, length of hospitalization, need for intensive care, incidence of systemic and local complications, necrosis, and pseudocyst formation were analyzed. Eight trials involving 557 patients were compared. The authors highlighted that lactated Ringer’s solution reduced severity, mortality, need of intensive care and systemic and local complications in acute pancreatitis.

In conclusion, this Special Issue, in addition to providing important scientific information on different topics, offers interesting new points of view on various pathologies and therapeutic applications.
